# Economic and social determinants of health care utilization during the first wave of COVID-19 pandemic among adults in Ghana: a population-based cross-sectional study

**DOI:** 10.1186/s12889-024-17912-4

**Published:** 2024-02-13

**Authors:** Martin Amogre Ayanore, Martin Adjuik, Roberto Ariel Abeldaño Zuñiga, Paul Amuna, Oliver Ezechi, Brandon Brown, Benjamin Uzochukwu, Nourhan M. Aly, Mir Faeq Ali Quadri, Bamidele Olubukola Popoola, Anthonia Omotola Ishabiyi, Passent Ellakany, Muhammad Abrar Yousaf, Jorma I. Virtanen, Folake Barakat Lawal, Eshrat Ara, Abeedha Tu-Allah Khan, Balgis Gaffar, Maha El Tantawi, Annie L. Nguyen, Moréniké Oluwátóyìn Foláyan

**Affiliations:** 1 Mental Health and Wellness Study Group, Ho, Ghana; 2 Department of Health Policy Planning and Management, Fred N. Binka School of Public Health, University of Health and Allied Sciences, Ho, Ghana; 3 Department of Epidemiology and Biostatistics, Fred N. Binka School of Public Health, University of Health and Allied Sciences, Ho, Ghana; 4 University of Sierra, Sur. Oaxaca, Mexico; 5 Fred N. Binka School of Public Health, University of Health and Allied Sciences, Ho, Ghana; 6 Department of Pediatric Dentistry and Dental Public Health, Faculty of Dentistry, Alexandria University, Alexandria, Egypt; 7 Department of Social Medicine, Population and Public Health, University of California, Riverside School of Medicine, Riverside, CA United States of America; 8 Department of Clinical Sciences, Nigerian Institute of Medical Research, Lagos, Nigeria; 9 University of Nigeria Nsukka (UNN) Enugu Campus, Nsukka, Nigeria; 10 Department of Family Medicine, Keck School of Medicine, University of Southern California, Los Angeles, CA United States of America; 11 Texas Tech University and Health Sciences Center, Texas, United States of America; 12 Department of Child Oral Health, University of Ibadan, Ibadan, Nigeria; 13 Department of Sociology, Florida Atlantic University, Boca Raton, Florida, USA; 14 Department of Substitutive Dental Sciences, College of Dentistry, Imam Abdulrahman Bin Faisal University, Dammam, Saudi Arabia; 15 Institute of Zoology, University of the Punjab, Lahore, Pakistan; 16 Faculty of Medicine, University of Bergen, Bergen, Norway; 17 Department of Periodontology and Community Dentistry, University of Ibadan and University College Hospital, Ibadan, Nigeria; 18 Government College for Women, Srinagar, Kashmir (J&K) India; 19 Department of Biological Sciences, Faculty of Allied Health Sciences, Superior University, Kot Araian, Raiwind Road, Lahore, Punjab Pakistan; 20 School of Biological Sciences, University of the Punjab, Quaid-e-Azam Campus, Lahore, Punjab Pakistan; 21 Department of Preventive Dental Sciences, College of Dentistry, Imam Abdulrahman bin Faisal University, Dammam, Saudi Arabia; 22 Department of Child Dental Health, Obafemi Awolowo University, Ile-Ife, Nigeria

**Keywords:** COVID-19, Health care utilization, Social determinants, Economic determinants, Ghana

## Abstract

**Background:**

The COVID-19 pandemic had socioeconomic effects in Africa. This study assessed the social and economic determinants of healthcare utilization during the first wave of COVID-19 among adults in Ghana.

**Methods:**

Information about individuals residing in Ghana was derived from a survey conducted across multiple countries, aiming to evaluate the impact of the COVID-19 pandemic on the mental health and overall well-being of adults aged 18 and above. The dependent variable for the study was healthcare utilization (categorized as low or high). The independent variables were economic (such as financial loss, job loss, diminished wages, investment/retirement setbacks, and non-refunded travel cancellations) and social (including food scarcity, loss of financial support sources, housing instability, challenges affording food, clothing, shelter, electricity, utilities, and increased caregiving responsibilities for partners) determinants of health. A multinomial logistic regression was conducted to identify factors associated with healthcare utilization after adjusting for confounders (age, gender, access to medical insurance, COVID-19 status, educational background, employment, and marital status of the participants).

**Results:**

The analysis included 364 responses. Individuals who encountered a loss of financial support (AOR: 9.58; 95% CI: 3.44–26.73; *p* < 0.001), a decrease or loss of wages (AOR: 7.44, 95% CI: 3.05–18.16, *p* < 0.001), experienced investment or retirement setbacks (AOR: 10.69, 95% CI: 2.60-43.88, *p* = 0.001), and expressed concerns about potential food shortages (AOR: 6.85, 95% CI: 2.49–18.84, *p* < 0.001) exhibited significantly higher odds of low healthcare utilization during the initial phase of the pandemic. Contrastingly, participants facing challenges in paying for basic needs demonstrated lower odds of low healthcare utilization compared to those who found it easy to cover basic expenses (AOR: 0.19, 95% CI: 0.06–0.67, *p* = 0.001).

**Conclusion:**

Economic and social factors were associated with low healthcare utilization in Ghana during the first wave of the pandemic. Investment or retirement loss and financial support loss during the pandemic had the largest effect on healthcare utilization. Further research is needed to understand the connection between concerns about food shortages, welfare losses during pandemics and healthcare utilization during pandemics in Ghana.

**Supplementary Information:**

The online version contains supplementary material available at 10.1186/s12889-024-17912-4.

## Introduction

The COVID-19 pandemic since its eruption in 2019 has had diverse social and economic impacts across the globe particularly in Africa [1, 2]. The COVID-19 pandemic has led to the disruption of the health system in Africa which is already weaker as compared to the healthcare systems in the global north. For instance, essential services related to HIV, tuberculosis, malaria, and maternal and child health have faced significant disruptions in Africa [3–5]. The economic and financial impact of the COVID-19 pandemic on Africa has been substantial, leading to a rise in poverty rates, heightened food insecurity, a decline in projected Gross Domestic Product, and a reversal of economic growth across most African nations [6]. This global crisis resulted in approximately one-third of workers losing their jobs, while two-thirds experienced a notable decrease in income ranging from 11.5 to 15.6% in 15 African countries [3]. These economic challenges have exacerbated the difficulties individuals face in accessing healthcare, a situation further complicated by the increased strain on health systems induced by the pandemic [6]. In response to these shocks, health systems underwent transformative changes aimed at saving lives and minimizing mortality rates [7, 8].

In Ghana, the government’s implementation of COVID-19 prevention measures during the initial two months of the pandemic led to disruptions in business operations, resulting in job cuts and losses [9]. Concurrently, the government increased spending to absorb economic shocks and address the social and economic needs of its citizens [10]. The populace also faced challenges related to delayed or limited access to health services, including access to antiretroviral therapy, newborn care diagnosis, and dental care services [11–14]. Additionally, Ghana had to manage a local outbreak of cerebrospinal meningitis [15].

While existing research acknowledges the negative impact of the pandemic on healthcare access in Ghana [16–19], there is limited understanding of the behavioral and socioecological determinants influenced by social and economic forces in the context of the COVID-19 pandemic. Building on previous studies that explored how socioecological factors shape healthcare utilization [20], this study emphasizes that an individual’s ability to seek and utilize healthcare during the pandemic is influenced by individual behavior and socioecological factors. Health behaviors, including healthcare-seeking, are intricately linked to beliefs and attitudes [20]. The study investigates economic and social determinants associated with healthcare utilization during the first wave of the COVID-19 pandemic among adults in Ghana.

Considering the severity of the pandemic and the implemented preventive measures such as lockdowns, social distancing, handwashing, and face mask usage, and guided by the socioecological model (SEM) of health behavior [20–22], the hypothesis posits that individuals facing significant social and economic losses amid the pandemic are less likely to utilize healthcare services. The study employed a behavioral and socioecological model to examine how social and economic factors shape population-level perceptions and access to healthcare during the COVID-19 pandemic. The model emphasizes the dual role of financial insecurity as both an economic and social determinant of health and well-being, with potential repercussions on mortality rates and mental health, particularly among vulnerable groups. This approach is crucial as pandemics often prompt immediate investments to mitigate immediate mortality and morbidity outcomes, overlooking the broader determinants of health that persist beyond the pandemic’s duration.

## Methods

### Ethical approval

Approval for the online data collection in this study was granted by the Human Research Ethics Committee at the Institute of Public Health, Obafemi Awolowo University, Ile-Ife, Nigeria (HREC No: IPHOAU/12/1557). Before engaging in the study, participants granted their consent by checking a designated box at the outset of the online survey.

### Design

This study was a cross-sectional study using a quantitative data collection approach. An online survey link was generated on the Survey Monkey® platform for the data collection.

### Sample size estimation

At the time of conducting the survey, there was a paucity of published evidence regarding the prevalence of healthcare utilization among specific groups or the general adult population in Ghana. To determine the required sample size, we employed the Cochrane formula [23]. Assuming a prevalence (p) of 0.5 for utilizing health care services during the first wave of the pandemic in Ghana, a 95% confidence level, and a margin of error of 0.0515, a sample size of 362 participants was deemed necessary. During survey administration, 364 individuals responded.

### Study participants’ recruitment

The survey started in July 2020 and ended in December 2020. The survey targeted individuals aged 18 years and above. For the recruitment process, we utilized non-probability respondent-driven sampling approaches. An online survey link was generated on the Survey Monkey® platform and disseminated to eligible participants. Initial contacts were encouraged to widely share the survey links within their networks, leveraging various social media platforms (*Facebook, Twitter, Instagram, and WhatsApp*) and email listservs. Detailed information on the study methodology has been previously reported in several publications [24–27].

### Data collection tool

Information was gathered using a standardized questionnaire that has been validated for global applicability [28]. The survey tool exhibited a commendable overall content validity index of 0.83. To prevent duplicate submissions, each respondent could participate only once, monitored by their unique internet protocol address. However, respondents had the flexibility to review and modify their answers to the questions before final submission. It’s important to note that there was no obligation for respondents to answer every question, allowing for voluntary participation.

#### Independent variables

##### Economic determinants

A series of five questions were employed to assess the economic factors related to COVID-19. Respondents were queried about the financial repercussions they faced due to the COVID-19 pandemic. They were provided with options to mark any applicable losses from the listed categories, including “loss of other sources of financial support,” “job loss or laid off,” “lost or reduced wages,” “investment/retirement loss,” and “travel-related cancellations that were not refunded.” The coding system assigned “0” when a response was not chosen and “1” when a response was selected. The set of questions demonstrated an internal consistency reliability with a Cronbach alpha score of 0.579.

##### Social determinants

Responses to a cluster of five questions were extracted from the database we designed for the online survey (Monkey® platform) and scrutinized to evaluate the social indicators amid the COVID-19 pandemic. Respondents were queried with the question: “Has the COVID-19 pandemic led to any of the following?” They were provided with the option to indicate ‘yes’ or ‘no’ to specific circumstances, including “loss of food,” “loss of sources of financial support,” “loss of housing,” “difficulty paying for basic needs (food, clothing, shelter, electricity, utilities),” and “the need to spend more time taking care of partners.” Responses were coded as “0” for ‘no’ and “1” for ‘yes.’ The set of questions in this section exhibited high internal consistency with a Cronbach’s alpha score of 0.832.

#### Dependent variable

##### Effect of social and economic determinants on healthcare utilization during the pandemic

A set of three questions was employed to gauge healthcare utilization during the pandemic. Respondents were presented with the query: “Has the COVID-19 pandemic led to any of the following?” The individual response questions included “unable to attend a healthcare provider’s appointment,” “unable to obtain medications that you take,” and “unable to afford medical care.” Responses were coded as “0” for ‘No’ and “1” for ‘Yes.’ A composite variable termed “effect of the pandemic on health care utilization” was derived and coded as “0, 1, 2 and 3.” For binary categorization for the responses, scores equal to or greater than the median were categorized as high healthcare utilization during the pandemic, while scores below the median were classified as low healthcare utilization during the pandemic. The set of questions in this section demonstrated strong internal consistency, with a Cronbach’s alpha score of 0.940.

#### Confounders

These comprised demographic variables: “age at last birthday,” “sex at birth (male, female),” “highest educational level (secondary, university, and postgraduate),” “employment status (unemployed, employed, student, retired),” “marital status (single, married, others [divorced or widowed]),” and “have medical health insurance (yes, no).” Additionally, the COVID-19 status may influence the utilization of health care services. The participants’ COVID-19 status was assessed through the following questions: “had a COVID-19 test, and the test was positive,” “experienced COVID-19 symptoms but did not test,” and “had a relative or close friend who tested positive for COVID-19”.

### Data analysis

Data analysis was conducted using STATA version 16.0®. Frequencies and percentages for all variables were computed. The Chi-square test assessed associations between binary dependent (healthcare utilization) and independent variables such as socio-demographic (age, employment status, marital status, educational level etc.) COVID-19 status (tested positive for COVID-19), social and economic determinants (job loss or laying off, lost or reduced wages, investment/ retirement loss). Multinomial logistic regression gauged the strength of the association between the outcome and predictor variables, adjusting for confounding variables. The significance level was established at α < 0.05.

## Results

### Participants’ sociodemographic profile and COVID-19 experiences

Table 1 shows that the respondents, numbering 364, had a mean (standard deviation) age of 31.6 (7.6) years, with 193 (53.0%) being male. COVID-19 positivity was reported by 18 participants (4.9%), while 22 (6.0%) faced job loss or layoffs, 65 (17.9%) experienced wage reduction or loss, 35 (9.6%) encountered investment/retirement loss, and 37 (10.2%) incurred losses from cancelled travel. Furthermore, 135 (37.1%) participants lost some financial support, 128 (35.2%) expressed concerns about potential food shortages, 8 (2.2%) experienced homelessness, and 97 (26.6%) struggled with paying for basic needs. A significant association was found between lost or reduced wages (*p*-value < 0.001), worry about food runouts (p-value < 0.001), loss of a source of financial support (*p*-value < 0.001) and difficulty paying for basic needs (*p*-value < 0.001) of the respondents and the healthcare utilization.

**Table 1:** Socio-demographic, COVID-19 status, social and economic determinants of related effect on healthcare utilization among adults in Ghana

Variables	Total *N* = 364 n (%)	Low healthcare utilization *N* = 216 n (%)	High healthcare utilization *N* = 148 n (%)	Chi-Square (***p***-value)
**Age [Mean (S.D)]**	31.6 (7.6)	31.1 (7.5)	32.4 (7.5)	
**Sex**				
Male	193 (53.0)	114 (52.8)	79 (53.4)	0.01 (0.910)
Female	171 (47.0)	102 (47)	69 (46.6)	
**Employment Status**				
Unemployed	40 (11.0)	25 (11.6)	15 (10.1)	0.19 (0.911)
Student	71 (19.5)	42 (19.4)	29 (19.6)	
Employed	253 (69.5)	149 (69.0)	104 (70.3)	
**Marital Status**				
Single	178 (48.9)	114 (52.8)	64 (43.2)	3.21 (0.201)
Legally married	161 (44.2)	88 (40.7)	73 (49.3)	
Other	25 (6.9)	14 (6.5)	11 (7.5)	
**Educational Level**				
Secondary	42 (11.5)	25 (11.6)	17 (11.5)	0.04 (0.982)
University	267 (73.4)	159 (73.6)	108 (73.0)	
Post-graduate	55 (15.1)	32 (14.8)	23 (15.5)	
**COVID-19 Status**				
**Tested positive for COVID-19**				
No	346 (95.1)	206 (95.4)	140 (94.6)	
Yes	18 (4.9)	10 (4.6)	8 (5.4)	0.11 (0.737)
**Experienced symptoms of COVID-19**				
No	322 (88.5)	195 (90.3)	127 (85.8)	
Yes	42 (11.5)	21 (9.7)	21 (14.2)	1.72 (0.190)
**Close friend tested positive for COVID-19**				
No	274 (75.3)	167 (77.3)	107 (72.3)	
Yes	90 (24.7)	49 (22.7)	41 (27.7)	1.19 (0.276)
**Social Determinants**				
**Job loss or laying off**				
No	342 (94.0)	205 (94.9)	137 (92.6)	
Yes	22 (6.0)	11 (5.1)	11 (7.4)	0.85 (0.357)
**Lost or reduced wages**				
No	299 (82.1)	206 (95.4)	93 (62.8)	
Yes	65 (17.9)	10 (4.6)	55 (37.2)	63.37** (< 0.001)
**Investment/ retirement loss**				
No	329 (90.4)	213 (98.6)	116 (78.4)	
Yes	35 (9.6)	3 (1.4)	32 (21.6)	41.37** (< 0.001)
**Travel-related cancellation**				
No	327 (89.8)	210 (97.2)	117 (79.1)	
Yes	37 (10.2)	6 (2.8)	31 (20.9)	31.75** (< 0.001)
**Economic Determinants**				
**Worry about food runouts**				
No	236 (64.8)	193 (89.4)	43 (29.1)	
Yes	128 (35.2)	23 (10.6)	105 (70.9)	140.05** (< 0.001)
**Loss of source of financial support**				
No	229 (62.9)	194 (89.8)	35 (23.6)	
Yes	135 (37.1)	22 (10.2)	113 (76.4)	164.78**(< 0.001)
**Loss of housing or homeless**				
No	356 (97.8)	211 (97.7)	145 (98.0)	
Yes	8 (2.2)	5 (2.3)	3 (2.0)	0.03 (0.854)
**Difficulty paying for basic needs**				
No	267 (73.4)	193 (89.4)	74 (50.0)	
Yes	97 (26.6)	23 (10.6)	74 (50.0)	69.58** (< 0.001)
**Spending more time taking care of partners**				
No	262 (72.0)	199 (92.1)	63 (42.6)	
Yes	102 (28.0)	17 (7.9)	85 (57.4)	106.96** (< 0.001)

* *p* < 0.05, ** *p* < 0.01, *** *p* < 0.001

Table 2 shows that participants experiencing reduced or lost wages (AOR: 7.44, 95% CI: 3.05–18.16, *p* < 0.001), investment/retirement losses (AOR: 10.69, 95% CI: 2.60-43.88, *p* = 0.001), concerns about food shortages (AOR: 6.85, 95% CI: 2.49–18.84, *p* < 0.001), and loss of financial support (AOR: 9.58, 95% CI: 3.44–26.73, *p* < 0.001) exhibited significantly higher odds of low healthcare utilization during the first wave of the pandemic. In contrast, participants facing difficulties in paying for basic needs demonstrated lower odds of low healthcare utilization compared to those not encountering such challenges (AOR: 0.19, 95% CI: 0.06–0.67, *p* = 0.001).

**Table 2:** Binary logistic regression analysis of the factors associated with low healthcare utilization among adults in Ghana

Variables	β	SE	COR (95%CI), ***p***-value	β	SE	AOR (95%CI), ***p***-value
**Social Determinants**						
**Lost or reduced wages**						
No			Ref.			Ref.
Yes	2.50	0.37	12.18 (5.95–24.95), < 0.001	2.01	0.46	7.44 (3.05–18.16), < 0.001
**Investment/retirement loss**						
No			Ref.			Ref.
Yes	2.97	0.61	19.59 (5.87–65.35), < 0.001	2.37	0.72	10.69**(2.60-43.88), **0.001**
**Travel-related cancellation**						
No			Ref.			Ref.
Yes	2.23	0.46	9.27 (3.76–22.88), < 0.001	0.91	0.56	2.49 (0.82–7.49), 0.106
**Economic determinants**						
**Worry about food runouts**						
No			Ref.			Ref.
Yes	3.02	0.29	20.49 (11.71–35.85), < 0.001	1.92	0.52	6.85*** (2.49–18.84), **< 0.001**
**Loss of source of financial support**						
No			Ref.			Ref.
Yes	3.35	0.30	28.47 (15.92–50.93), < 0.001	2.26	0.52	9.58*** (3.44–26.73), **< 0.001**
**Difficulty paying for basic needs**						
No			Ref.			Ref.
Yes	2.13	0.27	8.39 (4.89–14.39), < 0.001	-1.65	0.64	0.19* (0.06–0.67), **0.010**
**Spending more time taking care of partners**						
No			Ref.			Ref.
Yes	2.76	0.30	15.79 (8.73–28.57), < 0.001	0.64	0.44	1.89 (0.79–4.50), 0.151

Footnote: Observations = 364, Probability chil2 = 0.0000, Pseudo R^2^ = 0.5017. ^*^p < 0.05, ^**^p < 0.01, ^***^p < 0.001; β = Coefficient; SE = Standard error;

## Discussion

This study identified that encountering financial losses and facing the risk of food shortages significantly increased the likelihood of low healthcare utilization by 6.85 times during the COVID-19 pandemic. Conversely, participants experiencing difficulties in paying for basic needs demonstrated lower odds of low healthcare utilization during the pandemic. Predictive social factors such as lost or reduced wages and investment/retirement loss significantly heightened the odds of low healthcare utilization. These findings underscore the profound impact of broader economic and social determinants, such as financial losses and food scarcity, on individual access to and utilization of healthcare during the pandemic. Vulnerable individuals and those with lower socioeconomic status may have been disproportionately affected, facing challenges in accessing and utilizing healthcare services in Ghana during the pandemic.

We have established that financial insecurity acts as both an economic and social determinant influencing health and well-being. Specifically, experiences such as lost or reduced wages, investment/retirement loss, and loss of financial support during the pandemic were associated with an increased likelihood of low healthcare utilization among participants. In Ghana, instances of reduced wages [29], livelihood threats [30], and widespread job, financial, and investment losses [31] were frequently reported during the pandemic, driven by stay-at-home orders and business closures. Given that the private sector employs a significant proportion of permanent and temporary workers in Ghana [32], the pandemic’s disruptions to supply and demand chains resulted in extensive layoffs [33].

Our study findings underscore a pattern consistent with previous reports on global [34] and sub-Saharan African [35] levels, highlighting how financial insecurity exacerbates disparities in healthcare utilization in Ghana. Health needs can shift and escalate during pandemics [36, 37], and the inability to address underlying social and economic forces may contribute to reduced healthcare utilization. This has critical implications for population health, especially in communities already grappling with a substantial disease burden, potentially leading to a significant increase in mortality rates [38]. Moreover, it can impact the mental health and well-being of populations particularly vulnerable groups [39].

In a country like Ghana, where individual and household healthcare needs are primarily financed through out-of-pocket payments [40], the repercussions of financial losses such as lost or reduced wages and investment/retirement loss are significant for healthcare utilization [31]. This is directly tied to one’s capacity to afford and access healthcare services. Our study furnishes evidence suggesting that individuals experiencing financial insecurity during the COVID-19 pandemic may have encountered unmet healthcare needs. Although cash transfer schemes were introduced in Ghana during the pandemic by the Government to provide relief to small-scale businesses [41], their effectiveness in mitigating the impact of extensive layoffs by businesses remains unverified. Conducting empirical studies on catastrophic expenditures and welfare loss during and post-COVID-19 could offer valuable insights into how financial losses influenced healthcare utilization in Ghana.

The potential threat of financial insecurity, as highlighted in this study, may also be associated with a social risk factor contributing to the observed low utilization of healthcare during the COVID-19 pandemic and difficulty in paying for basic needs. The inability to afford necessities signifies a lack of purchasing power for individuals and households, hindering their ability to meet crucial medical healthcare needs during the pandemic. This situation may arise from financial challenges faced by individuals and households amid the pandemic. As a result, it becomes crucial to incorporate efforts to reduce financial losses into the priority-setting for pandemic response. These efforts could involve enhancing primary healthcare outreach programs, especially targeting communities that are disproportionately affected by financial insecurity.

Moreover, the results of the study reveal an association between the perception of food insecurity, specifically concerns about food stock depletion during the pandemic and low healthcare utilization among adult Ghanaians. A previous study suggested that food insecurity serves as a proxy measure for financial insecurity [42]. Consequently, the linkage between food insecurity and reduced healthcare utilization during the pandemic may be explained by underlying financial insecurity. These findings align with previous research indicating that food insecurity, stemming from economic and livelihood disruptions during the COVID-19 pandemic, correlates with adverse health outcomes [40, 41, 43].

The challenges posed by the pandemic, encompassing social, environmental, and ecological factors, contribute to a surge in food prices due to disruptions in the global food supply chain [44]. Consequently, individuals and households may prioritize spending on food over healthcare during the pandemic when resources are scarce. Further empirical studies are essential to comprehensively grasp the connection between concerns about food stock depletion and healthcare utilization during the COVID-19 pandemic, providing valuable insights for long-term health planning purposes.

### Strengths and weaknesses

One strength of this study lies in the diverse geographical distribution of participants across different residential locations in Ghana. The wide geographical distribution of participants has facilitated an understanding of the national impact of both social and economic determinants on healthcare utilization during the initial wave of the pandemic. Existing evidence published during this phase is scarce and often constrained in geographical scope [13, 30, 42, 45]. Although this study boasts a broader geographic representation, it is imperative to acknowledge that this study also has some limitations. For instance, the use of the convenience sampling method could have unintentionally excluded individuals lacking smartphones, internet access, or English proficiency from the study. This limitation hinders the generalizability of the study findings. Additionally, being a cross-sectional study, this study could not confidently establish causal relationships between the variables that were studied. Despite these inherent limitations, the study offers valuable insights that can inform pandemic response guidelines in the future.

This study, employing the socioecological model of health behavior, highlights the significant influence of broader economic and social determinants on individual healthcare access and utilization during the pandemic, with vulnerable populations and those of lower socioeconomic status disproportionately affected. It emphasizes the dual role of financial insecurity as both an economic and social determinant of health and well-being, with potential repercussions on mortality rates and mental health, particularly among vulnerable groups. In a context like Ghana, where out-of-pocket payments dominate healthcare financing, financial losses have profound implications for healthcare utilization. Efforts to address these challenges should be integrated into pandemic response priorities, including targeted outreach programs and initiatives to reduce financial losses. The study also underscores the association between food insecurity and reduced healthcare utilization, emphasizing the need for further empirical studies to elucidate this connection for effective long-term health planning.

## Conclusion

The first wave of the COVID-19 pandemic has exacerbated the pre-existing limited healthcare utilization in Ghana. Factors such as financial loss, reduced wages, investment loss, concerns about food shortages, and challenges in affording basic needs were predictive of decreased healthcare utilization among Ghanaian adults during the pandemic. These results underscore the significance of addressing financial and food insecurity during health emergencies, emphasizing the necessity for comprehensive national policies ensuring social, economic, and financial inclusion in pandemic control efforts. Further research is warranted to delve into the interplay between food shortages and welfare losses during the pandemic and their influence on healthcare utilization in Ghana.

### Electronic supplementary material

Below is the link to the electronic supplementary material.

Supplementary Material 1

## Data Availability

The datasets used and/or analysed during the current study are available from the corresponding author upon reasonable request.

## Abbreviations

AOR: Adjusted Odds Ratio
CI: Confidence Interval
COVID-19: Coronavirus disease
HIV: Human Immunodeficiency Virus
