# Supplementary material for: Economic and social determinants of health care utilization during the first wave of COVID-19 pandemic among adults in Ghana: a population-based cross-sectional study
Source: BMC Public Health. 2024 Feb 13;24:455. doi: 10.1186/s12889-024-17912-4 (PMC10865527; doi:10.1186/s12889-024-17912-4)
Supplement: Supplementary file 1 — Supplementary Material 1 [file 12889_2024_17912_MOESM1_ESM.docx]

**Additional File 1**: [supplementary file of participants demographics]

| **Variable** | **Frequency [N=364]** | **Percentage (%)** |
| --- | --- | --- |
| **Mean Age (S.D.)** | **31.6 (7.6)** |  |
| **Age Group** |  |  |
| 18-29 | 148 | 40.7 |
| 30-39 | 169 | 46.4 |
| 40+ | 47 | 12.9 |
| **sex** |  |  |
| Male | 193 | 53.0 |
| Female | 171 | 47.0 |
| **Employment Status** |  |  |
| Unemployed | 40 | 11.0 |
| Student | 71 | 19.5 |
| Employed | 253 | 69.5 |
| **Marital Status** |  |  |
| Single | 178 | 48.9 |
| legally married | 161 | 44.2 |
| Other | 25 | 6.9 |
| **Educational Level** |  |  |
| Secondary | 42 | 11.5 |
| University | 267 | 73.4 |
| Post-graduate | 55 | 15.1 |
| **Have medical insurance** |  |  |
| No | 60 | 16.5 |
| Yes | 304 | 83.5 |
| **Resort to alternative medical care services** |  |  |
| No | 314 | 86.3 |
| Yes | 50 | 13.7 |
| **Tested positive for COVID-19** |  |  |
| Yes | 18 | 5.0 |
| No | 346 | 95.0 |
| **Experienced the symptoms of covid-19** |  |  |
| Yes | 42 | 11.5 |
| No | 322 | 88.5 |
| **Close friend tested positive for covid-19** |  |  |
| Yes | 90 | 24.7 |
| No | 274 | 75.3 |
| **Knows someone who died from covid-19** |  |  |
| Yes | 90 | 24.7 |
| No | 274 | 75.3 |
| **Social determinants** |  |  |
| **Job loss or laying off** |  |  |
| No | 342 | 94.0 |
| Yes | 22 | 6.0 |
| **Lost or reduced wages** |  |  |
| No | 299 | 82.1 |
| Yes | 65 | 17.9 |
| **Investment/ retirement loss** |  |  |
| No | 329 | 90.4 |
| Yes | 35 | 9.6 |
| **Travel-related cancellation** |  |  |
| No | 327 | 89.8 |
| Yes | 37 | 10.2 |
| **Economic determinants** |  |  |
| **Worry about food runouts** |  |  |
| No | 236 | 64.8 |
| Yes | 128 | 35.2 |
| **Loss of source of financial support** |  |  |
| No | 229 | 62.9 |
| Yes | 135 | 37.1 |
| **Loss of housing or homeless** |  |  |
| No | 356 | 97.8 |
| Yes | 8 | 2.2 |
| **Difficulty paying for basic needs** |  |  |
| No | 267 | 73.3 |
| Yes | 97 | 26.7 |
| **Spending more time taking care of partners** |  |  |
| No | 262 | 72.0 |
| Yes | 102 | 28.0 |
| **Other means of accessing healthcare** |  |  |
| **In-person visit** |  |  |
| No | 288 | 79.1 |
| Yes | 76 | 20.9 |
| **Telehealth with video** |  |  |
| No | 358 | 98.4 |
| Yes | 6 | 1.7 |
| **Over the phone (no video)** |  |  |
| No | 335 | 92.0 |
| Yes | 29 | 8.0 |
| **Email or written communication** |  |  |
| No | 355 | 97.5 |
| Yes | 9 | 2.5 |
